# Molecular Dynamics Driven Design of pH-Stabilized Mutants of MNEI, a Sweet Protein

**DOI:** 10.1371/journal.pone.0158372

**Published:** 2016-06-24

**Authors:** Serena Leone, Delia Picone

**Affiliations:** Department of Chemical Sciences, University of Naples Federico II, Naples, Italy; London, UNITED KINGDOM

## Abstract

MNEI is a single chain derivative of monellin, a plant protein that can interact with the human sweet taste receptor, being therefore perceived as sweet. This unusual physiological activity makes MNEI a potential template for the design of new sugar replacers for the food and beverage industry. Unfortunately, applications of MNEI have been so far limited by its intrinsic sensitivity to some pH and temperature conditions, which could occur in industrial processes. Changes in physical parameters can, in fact, lead to irreversible protein denaturation, as well as aggregation and precipitation. It has been previously shown that the correlation between pH and stability in MNEI derives from the presence of a single glutamic residue in a hydrophobic pocket of the protein. We have used molecular dynamics to study the consequences, at the atomic level, of the protonation state of such residue and have identified the network of intramolecular interactions responsible for MNEI stability at acidic pH. Based on this information, we have designed a pH-independent, stabilized mutant of MNEI and confirmed its increased stability by both molecular modeling and experimental techniques.

## Introduction

Sweet proteins are a family of plant proteins able to elicit a taste sensation in humans. These proteins have been isolated from different tropical plants, and share no sequence or structure similarity, having in common only the vegetal origin and the physiological effect [1]. The most studied members of this family are monellin, from *Dioscoreophyllum cumminsii* [2], thaumatin, from *Thaumatococcus danielli* [3] and brazzein, from *Pentadiplandra brazzeana* [4]. All these proteins can interact with the sweet taste receptor, a heterodimeric G-protein coupled receptor formed by two subunits, T1R2 and T1R3, located on specialized taste cells on the tongue, palate and pharynx, which is also responsible for the perception of the sweet taste of small sugars and low molecular weight sweeteners [5–9]. Despite the similar effect, sweet proteins cannot bind to the same receptor site as small sweeteners because of their dimensions [10]. Hypotheses have been made to explain how such large molecules might interact with the T1R2-T1R3 heterodimer, among which the so called “wedge model”, which is, so far, one of the few theories able to describe and predict the functional binding of different sweet proteins to the same receptor [11–13]. According to this model, sweet proteins bind to a cleft on the exterior of the heterodimer, and the complementarity of surface shape and charge between the sweet protein and the receptor modulates their interaction [14,15]. This implies that only proteins that are correctly folded can activate the receptor [14–16], suggesting that structural stability is of great importance to preserve the protein function in different conditions. These effects have been widely investigated in the case of monellin, a prototypical sweet protein and one of the first members of this family to be isolated and characterized. Monellin is a small (~11 KDa), basic protein composed of two chains arranged in a cystatin like fold [17,18]. Previous studies have shown that point mutations that slightly modify the three dimensional shape of the protein surface can greatly reduce the physiological effect, even when the overall fold is maintained [16,19–21]. Accordingly, the loss of structural integrity that follows thermal denaturation or pH variations also results in a loss of effect. Native monellin loses its activity when heated above 50°C, due to disruption of the heterodimeric structure. To increase thermal stability, single chain derivatives have been designed, joining the two subunits directly together [22] or through a dipeptide Gly-Phe linker [23]. These proteins are more stable than the parent protein and, at acidic pH, they regain sweetness even after few minutes boiling in water solution [22]. The reversibility and reproducibility of thermal and/or chemical unfolding of single chain monellins has allowed for their use as model systems for folding and unfolding studies [24–35].

In addition to temperature, single and double chain monellins also show a marked stability dependence from pH. Analyses of the unfolding kinetics have proven that both variants are strongly destabilized by the increase of pH from 4.0 to 10.0. Aghera et al. demonstrated that this effect is due to a single glutamic residue [24,25] buried at the C-terminal region of the helix (E24 according to Aghera's nomenclature, E23 according to structure 1FA3 of the Protein Data Bank, lacking the start methionine, hereafter used in the text [36]). As a consequence of its position in a hydrophobic pocket, the side chain of E23 exhibits a high pK_a_, a phenomenon often observed when ionizable residues are located in the interior of the protein fold, which often leads to a marked pH-dependent stability [37–41]. From the analysis of the folding kinetics, Aghera et al. estimated that the pK_a_ of the side chain of E23 in the native state is approximatively 7.5. The abrupt change in pK_a_ (to ~4.5 for the exposed glutamic side chain) that occurs with unfolding is the cause of the observed destabilization of MNEI at neutral to alkaline pHs. Destabilization of the native state of globular proteins can also lead to other unfavorable or uncontrollable phenomena, among which aggregation [42] and former studies have indeed pointed out the tendency of MNEI to form fibrillar aggregates, a tendency accentuated by increasing pHs or temperatures [32,33,43–46]. All these phenomena reduce the potential of MNEI for industrial applications, and should therefore be resolved before the protein can be actually employed in large scale processes. On the basis of previously acquired structural knowledge, we have tried to understand the factors involved at the atomic level in MNEI stabilization. We have evaluated the theoretical pK_a_ of E23 side chain using Multi Conformation Continuum Electrostatics calculations on various available experimental structures, and performed molecular dynamics simulations at different pHs and temperatures, in order to define the contribution of E23 to the fold stability. We have then designed a stabilized mutant, MNEI-E23Q, in which replacement of the glutamic residue with a glutamine allows the preservation of the network of interactions of the native state in a pH-independent manner. Increased stability of such mutant, as predicted by MD simulations, has been then confirmed by thermal unfolding studies using CD spectroscopy.

## Materials and Methods

### Molecular structures and continuum electrostatic calculations

Available experimental structures for MNEI used in this paper have PDB-IDs 1FA3, NMR resolved [36] and 2O9U, X-ray determined [47]. Residues numbering follows 1FA3 structure, lacking the start methionine. Protein secondary structure elements are referred to within the text using the following nomenclature: *Nt* (1–4), *β1* (4–6), *Lα1* (6–9), *α1* (10–27), *Lα2* (28–34), *β2* (35–48), *L23* (49–54), *β3* (55–64), *L34* (65–68), *β4* (69–78), *L45* (79–83), *β5* (84–90), *Ct* (91–96). Inspection and manipulation of the structures was performed with UCSF Chimera v. 1.9 [48]. Multi-conformation continuum electrostatic (MCCE) calculations were performed with MCCE 2.7 [49–51], using a dielectric constant for the protein ε_P_ = 4 and a salt concentration of 150 mM.

### MD simulations

Molecular dynamics were performed on model 1 of 1FA3 as the starting structure. Dynamics and trajectory analysis were performed with the software package GROMACS 4.6.4 [52–54] using the AMBER ff03 force field [55]. Structures with protonated and ionized side chain for E23 (MNEI-GLH and MNEI-GLU) and for mutant MNEI-E23Q were generated and immersed in a rhombic dodecahedron box with periodic boundary conditions and solvated with TIP3P water molecules [56]. The appropriate number of Cl^-^ ions was added to neutralize the system. Long-range electrostatic interactions were treated with the particle-mesh Ewald method with a grid spacing of 0.12 nm [57], and a long range cutoff of 8 Å was used. The LINCS algorithm was used to constrain bond lengths and a 2 fs time step was used [58].

For the simulations at room temperature, the molecules were submitted to initial energy minimization with 5000 steps of steepest descent, followed by 100 ps NVT and 300 ps NPT equilibration at 300 K with position restraints. For the high temperature simulations, after solvent relaxation, initial velocity distributions were generated at 300 K, followed by 50 ps equilibration at this temperature. Temperature was then increased step-wise over 40 ps to 473 K and the system temperature was further equilibrated for an additional 10 ps. Production runs were performed for 10 ns with a 2 fs step. Temperature and pressure coupling were obtained with the v-rescale [59] and the Parrinello-Rahman [60]algorithms, respectively. Surface accessibilty of the residues was calculated with the program g_sas from the GROMACS package was used. Relative surface accessibility was estimated by normalizing the values obtained over time to the maximum surface accessibility as calculated in the tripeptide Gly-X-Gly [61].

### Proteins expression and purification

The synthetic gene encoding for MNEI-E23Q was purchased from Eurofins Genomic and cloned in the pET22b+ vector (Novagen) within the *Nde I* and *BamH I* restriction sites. Vector pET22b+ carrying the gene encoding for MNEI was the same as previously described [36,44]. To express the recombinant proteins, cells of *Escherichia coli* BL21(DE3) were transformed with said plasmids; cells were cultured in 1L of LB medium containing 100 mg/L ampicillin. Protein expression was induced at 0.6 OD with 5 mM lactose and cells were harvested by centrifugation (4°C, 3000 x *g*, 20 min) after 20 h induction at 25°C, washed with cold PBS and stored frozen until extraction. Purification was achieved in a one-step procedure as described [62]. Briefly, cell lysates in 50 mM sodium acetate at pH 5.5 were applied to a DEAE-Sepharose (20 mL, GE Lifesciences) connected in series to a Macro-Prep High S (15 mL, Bio-Rad). Proteins were then eluted from the Macro-Prep High S with 2 CV of 100 mM NaCl in 50 mM Sodium Acetate, pH 5.5. The protein containing fractions were desalted by Size Exclusion Chromatography on a Sephadex G-25 column (GE Lifesciences, 2.5 x 26.5 cm, 130 mL) in 75 mM AcOH at 5 mL/min and freeze-dried; purity was assessed by SDS-PAGE. Protein yield was estimated by UV absorbance and was on average 50 mg for both proteins per liter of culture.

### CD spectroscopy

Protein fold integrity was assessed by circular dichroism (CD) spectra recorded on a Jasco J-715 spectropolarimeter equipped with a Peltier temperature control system (PTC-348WI). Molar ellipticity per mean residue [θ] in deg cm^2^ dmol^−1^ was calculated from the equation: [θ] = [θ]_obs_ mrw/(10 × l × C), where [θ]_obs_ is the ellipticity measured in degrees, mrw is the mean residue molecular weight of the protein (Da), C is the protein concentration in g/mL and l is the optical path length of the cell in cm. Cells of 0.1 cm path length were used. CD spectra were recorded with a time constant of 4s, a 2 nm band width and a scan rate of 20 nm/min, and the signal was averaged over three scans and baseline corrected by subtracting a buffer spectrum. Spectra were recorded in 20 mM phosphate buffer at pH 3.5, 5.1 and 6.8 and in 20 mM Tris-HCl at pH 8.0. A concentration of 0.25 mg/mL protein was used for each sample, as determined by UV absorbance at 280 nm prior to CD measurement.

Thermal denaturation experiments were recorded following the signal at 215 nm while varying the temperature from 30 to 95°C at a rate of 1°C/min. For each condition, three independent measures were performed. Experimental points were fitted to a Boltzmann curve, and fraction of unfolded protein (*f*_*u*_) was calculated according to the formula:

$$f_{u}\;=\;\frac{\theta_{f}-\theta }{\theta_{f}-\theta_{u}}$$

where θ_f_ and θ_u_ are the CD signal of the folded and unfolded state, respectively and θ is the CD signal at each temperature from the fitted curve.

## Results

### Continuum Electrostatic pK_a_ determination

**Fig 1:**
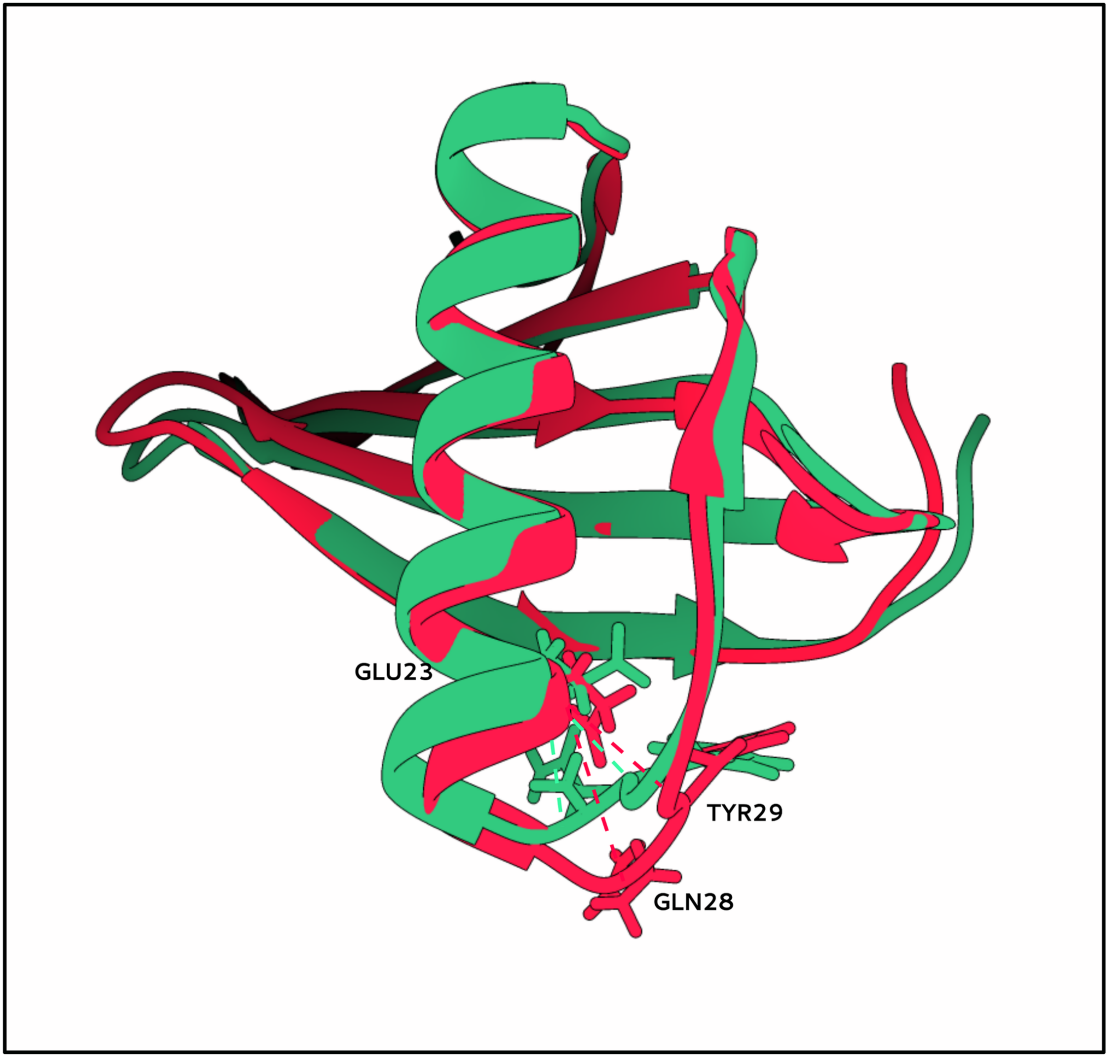
Comparison of available experimental structures of MNEI. Overlay of experimental structures of MNEI derived from NMR (1FA3, green) and X-rays crystallography (2O9U, red). The image focuses on loop Lα2 and on the distances E23-Q28 and E23-29 (dotted lines), defining the opening of the loop and allowing for water penetration from the bulk of the solvent.

MNEI is a small, globular protein that does not contain histidine residues, but exhibits a pH dependent behavior at near physiological values. Aghera et al. had already associated this phenomenon to residue E23 and, on the basis of the unfolding energies, they had estimated that the pK_a_ of its side chain in the native state was approximately 7.5 [24]. As a rigorous titration of the pK_a_ of E23 side chain is still missing, we tried to provide a theoretical estimation of the value expected on the basis of available experimental structures by Multi Conformational Continuum Electrostatics (MCCE [51]), to understand whether the observed behavior could be predicted. MCCE calculations explicitly simulate side chain motions of amino acids by Monte Carlo methods, while leaving the backbone unaffected. By choosing the MCCE approach, our aim was to minimize the differences introduced by different side chains orientations in the experimental structures in the continuum electrostatic pK_a_ calculation. Both the crystal and NMR structures of MNEI (PDB-ID 2O9U and 1FA3) [36,47] were used. Since the NMR derived structure contains a conformational ensemble, we performed the calculations on each element of the cluster. When starting from the crystal structure, we obtained a theoretical pK_a_ value of 5.2, lower by more than two units than the approximate experimental result [24] and closer to the typical value of 4.5 for the exposed glutamic side chain. Calculations on structure 1FA3 resulted in pK_a_ values ranging from a minimum of 5.9 in model 9 to 8.9 in model 15. By comparison, the average value of pK_a_ for the neighboring E22 side chain was 4.7 over the 20 structures. Structures 2O9U and 1FA3.15 represent therefore the extremes of the theoretical pK_a_ interval predictable for MNEI. A comparison of region 21–33 in the two models, corresponding to the C-terminus of the helix and to the facing portion of loop *Lα2*, shows a RMSD between heavy atoms of only 1.7 Å. In the crystal structure, the region appears slightly wider than in the NMR ensemble (Fig 1) as indicated by the distances between the carboxyl oxygens of E23 and the carbonyl oxygen of Y29 (5.7 Å and 6.2 Å in 1FA3.15 and 2O9U, respectively) and E23(O) and Q28(N) (2.9 and 4.7 Å). The crystal structure also shows that a molecule of water (W17 in the PDB) is stably bound and buried in correspondence of the loop, but this water molecule was not observed in solution NMR studies with paramagnetic probes of the hydration of MNEI surface [63]. NMR and crystallization experiments were performed at different pHs (2.9 and 5.6, respectively). The occurrence of water in the crystal structure might be a consequence of forced packing interactions, leading to the underestimation of E23 pK_a_. On the other hand though, the experiments might be capturing the situations occurring in different conditions. Taken together, the results suggest that loop *Lα2* is provided with a certain flexibility, and that slight displacements, even thermal motions, can produce significant changes in the accessibility of the protein interior to water, with consequent changes in the polarizability experienced by E23 side chain, abrupt changes of its pK_a_ and significant variations of MNEI stability.

### MD simulations of different protonation states

**Fig 2:**
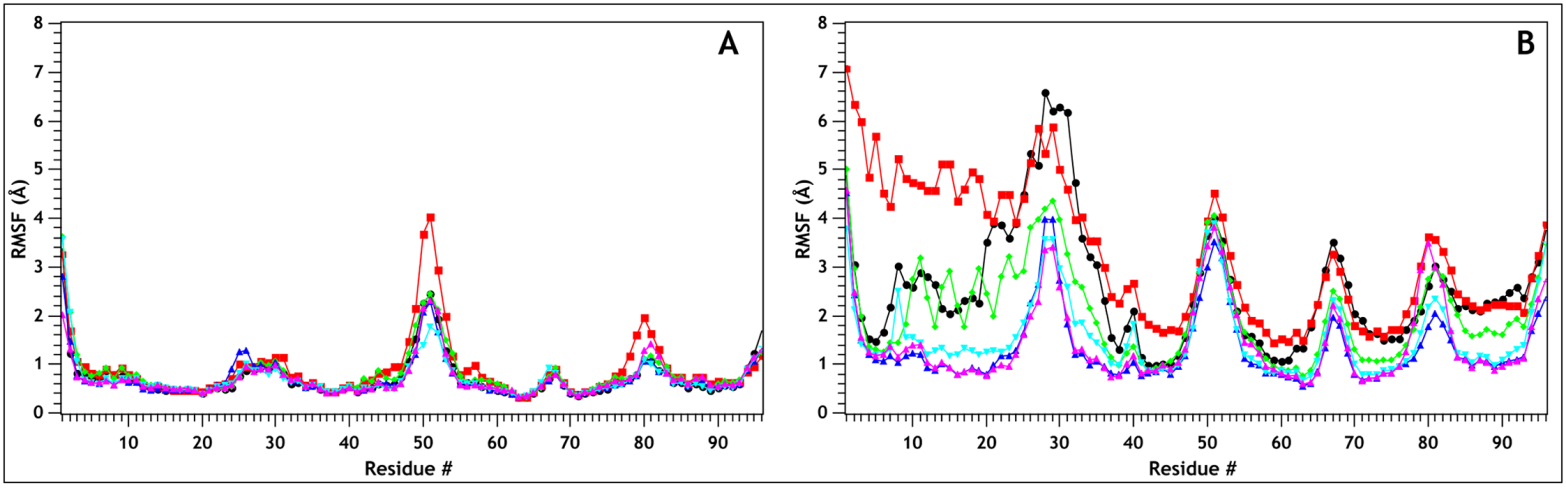
C_α_-Root Mean Square Fluctuation for the different MD simulations. The figure shows the C_α_-RMSD for the various runs at 300 K (A) and 473 K (B) on MNEI-GLU (MNEI-GLU_1, black; MNEI-GLU_2, red; MNEI-GLU_3, green) and MNEI-GLH (MNEI-GLH_1, blue; MNEI-GLH_2, cyan; MNEI-GLH_3, magenta). In all the simulation at room temperature, the structure remains stable and the highest flexibility is observed at the loops between the secondary structure elements. When the temperature is increased, the N-terminal portion exhibits a wider displacement from the experimental fold if E23 side chain is deprotonated.

The values obtained for the pK_a_ of E23, either from existing literature and from the NMR structures, point toward the occurrence of a switch in its protonation state at neutral to mildly alkaline pHs. This is indeed the pH range at which irreversible denaturation and precipitation of MNEI has been observed [14,31,43,44]. As previously noted, MNEI does not contain histidines and E23 is the only residue that can undergo a protonation change in this range of pH. To investigate the consequences at the atomic level of this event, we performed three independent 10 ns MD simulations for each of the two protonation states of E23 on PDB model 1FA3 (MNEI-GLU/ MNEI-GLH). The simulations were carried out at room temperature (300 K) and high temperature (473 K), to accelerate potential local unfolding events in consequence of different stabilities, according to well established protocols [64–72]. Although this approach may not allow exhaustive sampling of all the conformations assumed by the partially unfolded protein, which would require, for instance, longer simulations at lower temperature [73,74], it is nonetheless suitable to highlight the weak spots within the structure where unfolding initiates, suggesting potential sites for genetic manipulations. The simulations at room temperature confirmed the structure stability at every pH, in accordance with experimental data [24,43,44]. The C_α_-RMSD plot for each simulation reaches a plateau within 2 Å from the NMR structure (S1 Fig), whereas the plot of the RMS fluctuation for each residue shows that the regions of higher flexibility are localized, as expected, at the N- and C-termini and at the loops between the strands (Fig 2A). The helix appears stably positioned in the *β*-grasp and the mobility of its C-terminus, where E23 is located, is substantially unaffected by its protonation state. Upon increase of the simulation temperature to 473 K, the structure of MNEI-GLH is substantially unaffected: the RMSD remains within 3 Å of the NMR structure for the first 7 ns of the simulation and unfolding begins only at the end of the MD run (S1 Fig). This is consistent with the experimentally observed thermostability at acidic pH of MNEI [24,43,44]. When E23 is deprotonated, the RMSD diverges, reaching values above 5 Å after only 4 ns, and the protein proceeds toward fast unfolding. A plot of the RMSF shows that N-terminal of the protein, up to residue 40, becomes increasingly mobile (Fig 2B), with the helix being displaced from its original position up to 7 Å. The remaining portion of the protein is more stable, but either the loops and the *β*-strands have higher mobility compared to MNEI-GLH, suggesting that the destabilization is conveyed through the entire protein structure.

**Fig 3:**
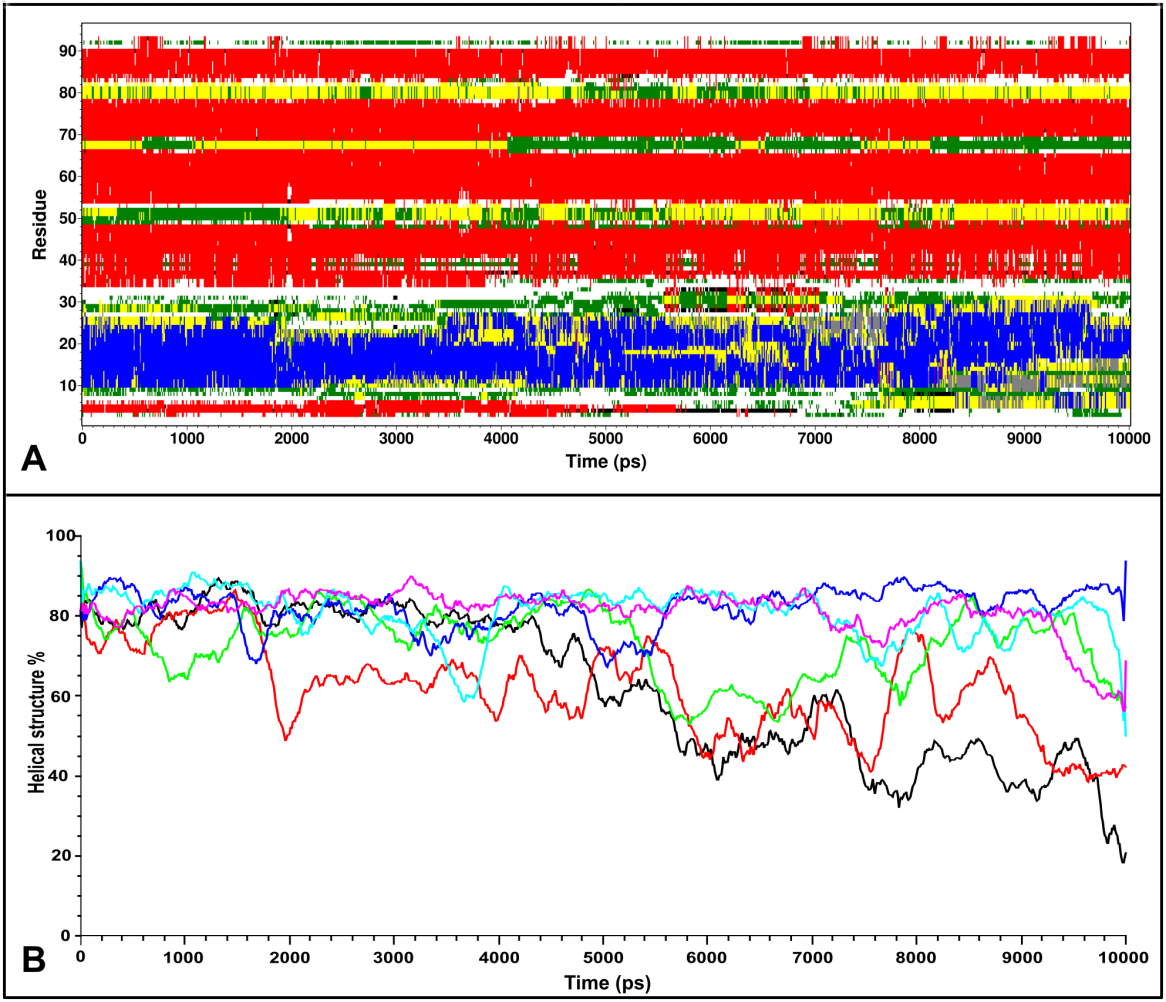
Time evolution of secondary structure. The figure displays the change over time of secondary structure for run MNEI-GLU_473_2 (A) and the percentage of residual helical structure (B) for MNEI-GLU (MNEI-GLU_1, black; MNEI-GLU_2, red; MNEI-GLU_3, green) and MNEI-GLH (MNEI-GLH_1, blue; MNEI-GLH_2, cyan; MNEI-GLH_3, magenta). Secondary structure elements were calculated with DSSP and are color coded (blue, α-helix; red, β-sheets; yellow, turns; green, bend; grey, 3-helix). During high temperature simulations, MNEI-GLU consistently looses secondary structure of portions of the helix, which remains structured for as little as 20% of the starting value. Residues in proximity of the *Lα2* loop become partially unstructured after only 2 ns. Secondary structure plots for all the simulations are reported in the supplementary material.

**Fig 4:**
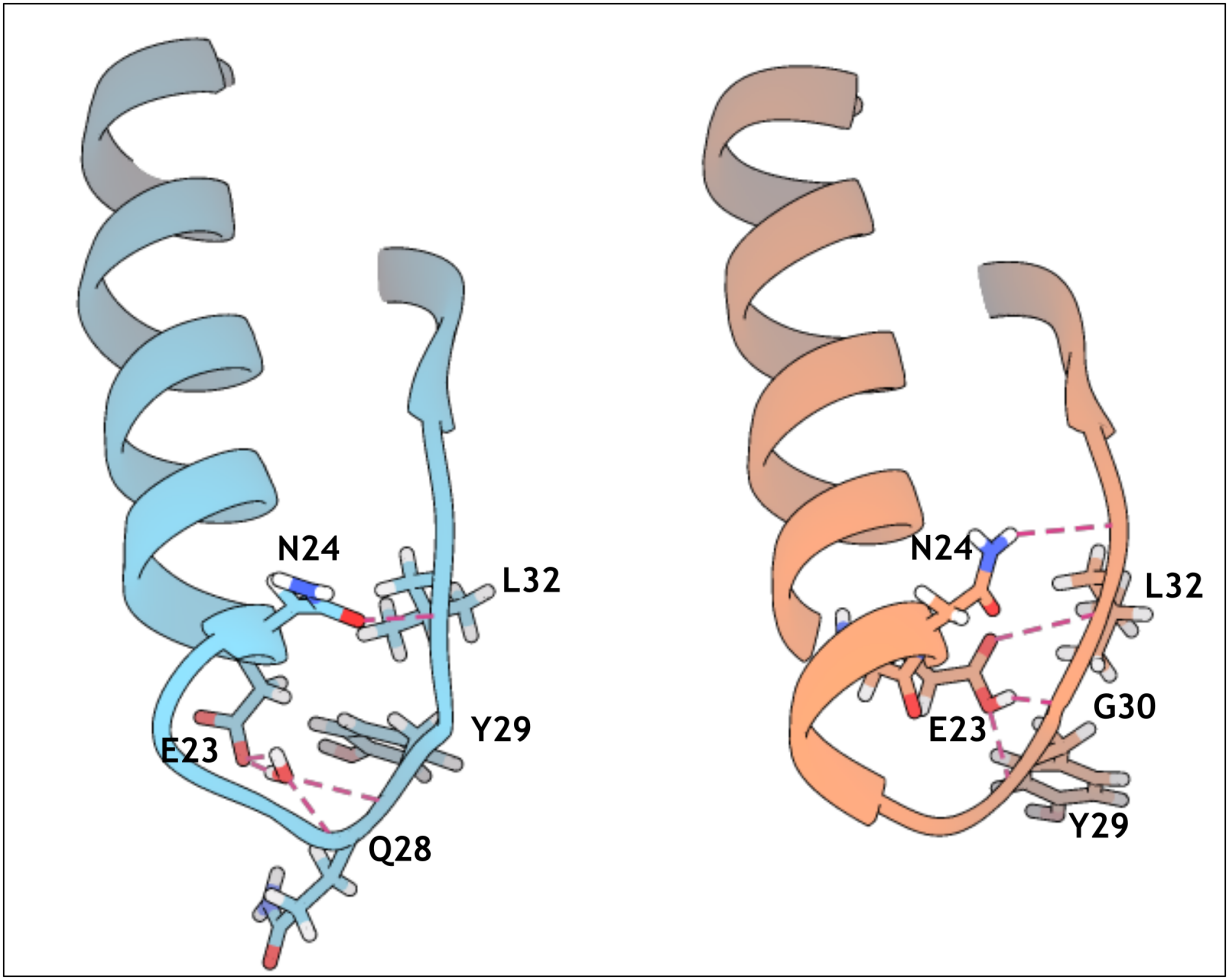
Main stabilizing interactions from MD simulations. Snapshots of MD trajectories at 300 K describing the principal non-secondary structure interactions in MNEI-GLU (blue) and MNEI-GLH (pink). Penetration of a water molecule in the loop does not compromise the stability of the structure at room temperature, but disrupts the zipping network of H-bonds occurring when E23 side chain is protonated, leading to faster unfolding of the helix in the simulations at 473 K.

To understand the nature of these motions and their effect on the fold, we performed secondary structure calculations over time with DSSP [75,76]. Analysis of the simulations at 300 K shows no substantial deviation of secondary structure elements from their experimental description, a situation also observed for MNEI-GLH at 473 K (S2 and S3 Figs). When E23 side chain is ionized, though, we observe a partial disruption of the secondary structure starting as early as 4 ns, which explains the observed RMSD increase. Unfolding occurs to different extents in the three independent simulations, but it always involves residues at the C-terminal portions of the helix, as exemplified in Fig 3A, which shows the DSSP plot for one of the high temperature simulations on MNEI-GLU. While the helical structure is only marginally lost in the 473 K simulations on MNEI-GLH, the percentage of residual helical structure drops up to 20% of the starting value in MNEI-GLU (Fig 3B). To understand the contribution of E23 to the protein stability, we evaluated the *intra*-molecular interactions occurring in the native state from the simulations at 300 K. The protonated side chain can form a very stable H-bond with the carbonyl oxygen of G30: the average distance between the carboxyl in the side chain of E23 and G30(O) is 2.78 Å and the occurrence of such bond is 99.4% over the three simulations. By tightening the loop between the helix and *β*2, several non-secondary structure H-bonds between side chains are formed and show high occupancy, adding stability to the fold, as reported in Table 1. Despite being situated on a flexible loop, E23 remains stably buried throughout the room temperature simulations. A calculation of the relative surface accessibility (RSA) shows that on average E23 side chain is exposed to the solvent for only 4%. This value is well below the 20–25% threshold typically used to define solvent accessible residues in two states models [77–80]. This means that the flexibility of *Lα2* is not enough to expose E23 side chain and the side chain can remain protonated (S3 Fig). In comparison, repulsive forces in MNEI-GLU result in a slight opening of the loop and consequent partial exposure of E23, which has an average RSA at 300 K around 20%, indicating that over time the residue can come in contact with the solvent [77,80]. At lower temperatures, the charge on the side chain of E23 can still be stabilized through hydration. Indeed, in each of the simulations at room temperature, a water molecule penetrates in the space at the C-terminal of the helix after few picoseconds, mediating the interactions between E23 and the amide protons of Y29 and Q28. This water mediated stabilization prevents the formation of the contacts observed in MNEI-GLH, but does not compromise the protein fold in the low temperature simulations. Nonetheless, in high temperature simulations, this results in faster unfolding, as evident in the secondary structure prediction plots (S3 Fig). The most relevant non-secondary structure interactions of the two states and their occurrence over time are reported in and Table 1 and represented in Fig 4.

**Table 1:** Hydrogen bonds from MNEI simulations.

	GLU1	GLU2	GLU3	GLH1	GLH2	GLH3
**ARG84(H**_**η**_**)—ASN14(O**_**δ1**_**)**	*35*.*7*	*18*.*8*	*48*.*5*	*47*.*9*	*37*.*2*	*24*.*5*
**LEU32(H)—GLU23(O**_**ε**_**)**				*39*.*0*	*43*.*0*	*36*.*3*
**ARG31(H**_**η1**_**)—ASN24(O)**				*13*.*3*	*17*.*4*	*37*.*8*
**TYR29(H)—GLU23(O**_**ε2**_**)**				*67*.*1*	*83*.*7*	*81*.*6*
**ASN24(H**_**δ**_**)—LEU32(O)**		*33*.*7*		*50*.*8*	*52*.*2*	*56*.*0*
**GLU23(H**_**ε2**_**)—GLY30(O)**				*99*.*7*	*99*.*3*	*99*.*1*
**GLN13(H**_**ε**_**)—ILE8(O)**	*21*.*8*	*11*.*3*	*38*.*8*	*76*.*6*	*65*.*8*	*77*.*4*
**THR12(H**_**γ1**_**)—ASP7(O)**	*49*.*4*	*36*.*8*	*56*.*3*	*86*.*9*	*89*.*6*	*94*.*2*
**LEU32(H)—ASN24(O**_**δ1**_**)**	*87*.*3*	*90*.*1*	*92*.*0*			
**GLN28(H**_**ε2**_**)—GLU23(O**_**ε2**_**)**	*16*.*9*					
**GLY9(H)—THR12(O**_**γ1**_**)**	*38*.*1*	*65*.*5*	*28*.*6*			

Principal non-secondary structure H-bonds in the *Lα1* loop and percent occupancy from the three MD simulations at 300 K on MNEI-GLU and MNEI-GLH.

### Construction of the MNEI-E23Q mutant

**Fig 5:**
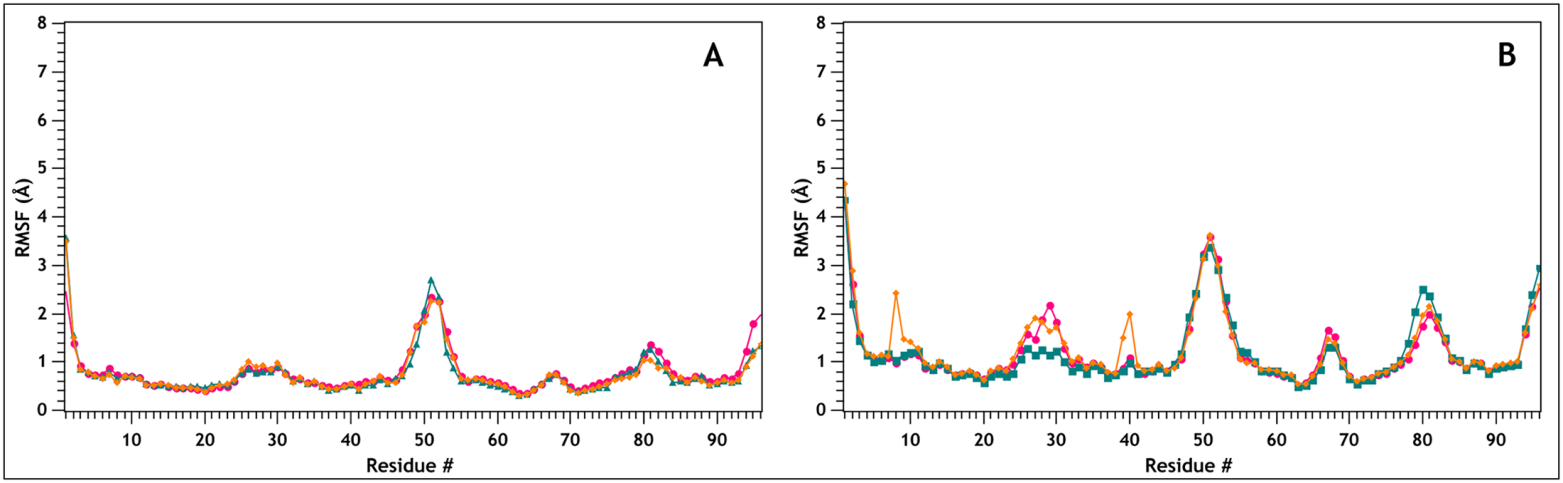
C_α_-Root Mean Square Fluctuation of MNEI-E23Q. C_α_-Root Mean Square Fluctuation for the three independent 10 ns MD simulations at 300 K (A) and 473 K (B) on MNEI-E23Q (MNEI-E23Q_1, pink; MNEI-E23Q_2, green; MNEI-E23Q_3, orange). Increasing the temperature of the MD runs leads to more flexibility at the loops, but stability is comparable to the simulations at room temperature.

**Fig 6:**
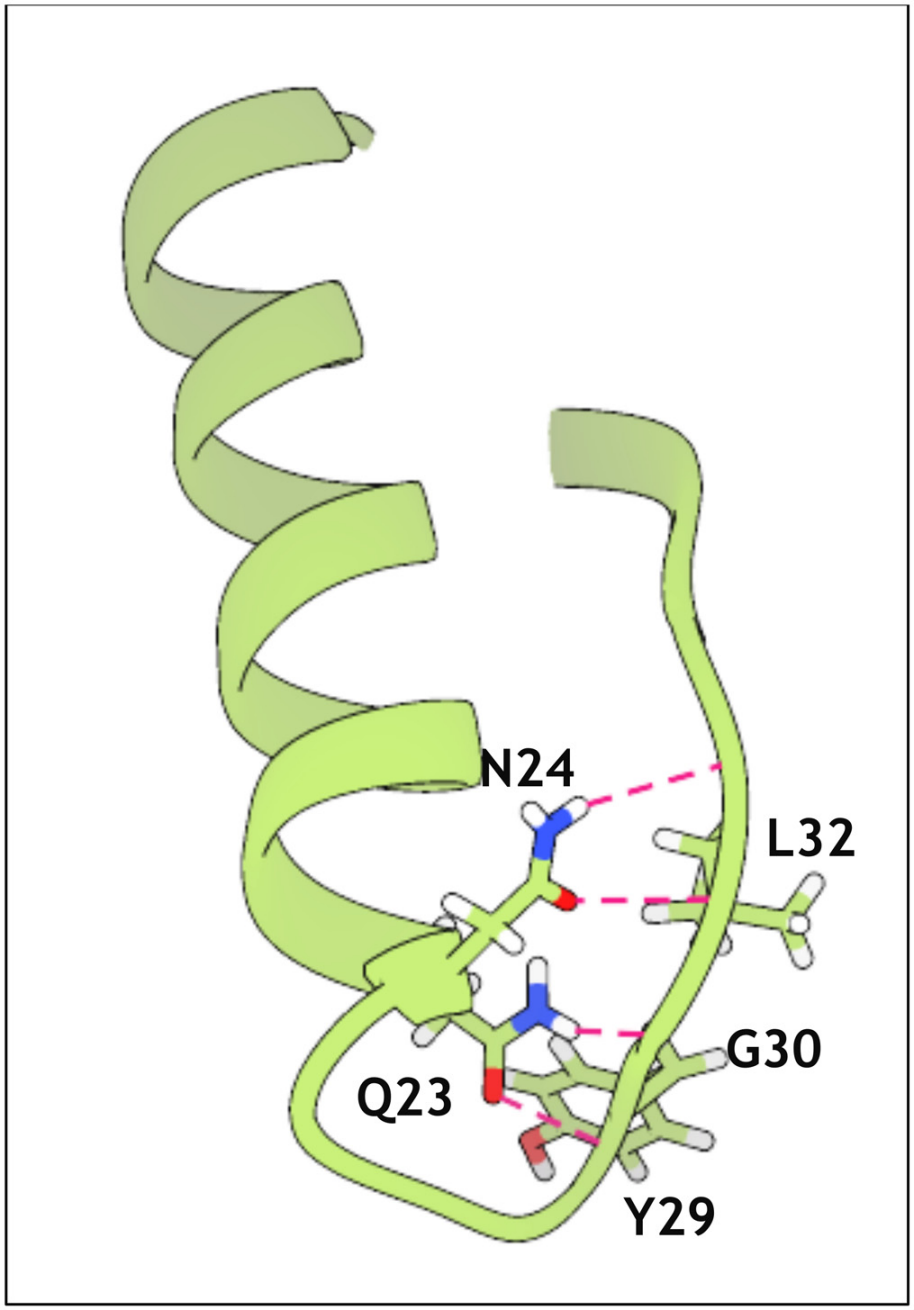
Stabilizing interactions in MNEI-E23Q. Snapshot of MD trajectory at 300 K describing the principal non-secondary structure interactions in MNEI-E23Q. These hydrogen bonds recreate the network stabilizing MNEI-GLH, allowing for the same thermal stability at any pH.

**Fig 7:**
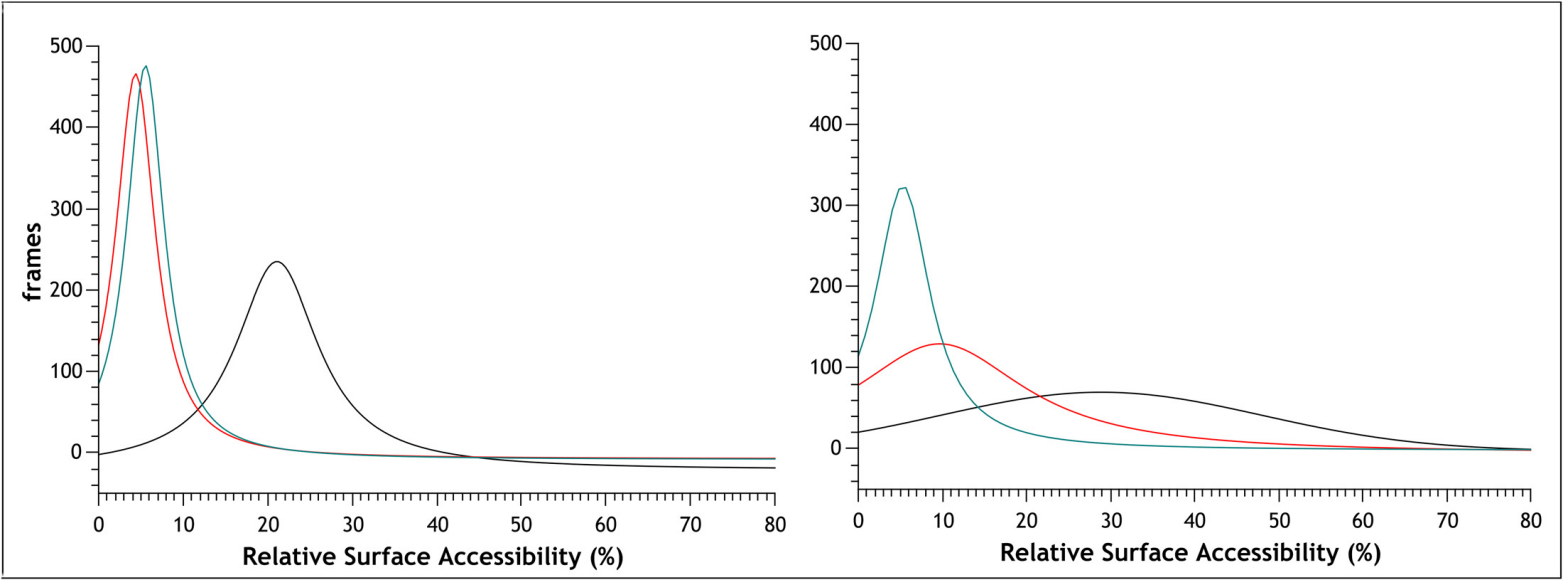
Relative Surface Accessibility from MD simulations. Distribution of relative surface accessibility of E23/Q23 in the three MD simulations for MNEI-GLU (black), MNEI-GLH (red), MNEI-GLN (green) at 300 (A) and 473 K (B). The figure shows that residue 23 remains stably buried in loop *Lα2* in the simulations on MNEI-GLH and MNEI-E23Q. When the residue is deprotonated, average RSA is around 20%, a value that represents partial accessibility to the bulk solvent. At high temperature, partial unfolding of the helix C-terminal completely exposes E23 in MNEI-GLU, whereas the residue remains inaccessible to the bulk water in MNEI-GLH and MNEI-GLU.

The strong interaction between the carboxyl proton of E23 and the carbonyl oxygen in G30 contributes to maintain the integrity of the fold in MNEI by sealing a network of stabilizing H-bonds that ensures thermal stability. In order to recreate these interactions while removing pH dependancy, we analyzed the model of mutant MNEI-E23Q. As previously observed, removal of the ionizable side chain of E23 eliminates the dependancy of the stability from pH [24]. At the same time, the glutamine side chain should allow the formation of the favorable network of interactions observed in MNEI-GLH, likely increasing thermal stability at alkaline pH. Moreover, compared to the described alanine mutant [24], the presence of a side chain of comparable length in Q23 would avoid small distortion in the three dimensional structure, thus preserving the sweetness of MNEI. We know in fact that even slight deformations in the protein shape and local flexibility, such as those introduced by a single point alanine mutation, are able to impair the interaction with the sweet taste receptor and reduce the sweetness [81,82]. Again, we ran three independent simulations at 300 and 473 K. Analysis of the RMSD and RMSF plots shows that the structure is very stable at room temperature, with the area of highest flexibility localized in the *L45* loop (residues 78–83), in accordance with what observed for the native structure. The mutant remains stable even after 10 ns simulations at 473 K, displaying minimal RMSD from the starting structure on the observed time scales even at such high temperatures (not shown). RMSF plots (Fig 5) show that, even at high temperature, molecular motions are limited, and deviations from the experimental structure remain below 2 Å. Loop *L23* and, in general, the loops between the β-strands show comparable mobility as in MNEI-GLH. Moreover, loop *Lα2* is globally more rigid than in the structures containing the glutamic acid in either protonation state. Secondary structure analysis confirms that the α and β elements are stable throughout the simulations (S2 and S3 Figs). In each of the three 300 K simulations, the side chain of Q23 establishes an interaction with the backbone oxygen of G30, helping retrace the stabilizing H-bonds between the helix and *β2* of MNEI-GLH, thus incrementing resistance to unfolding at any pH. These interactions are listed in Table 2 and depicted in Fig 6. Water does not penetrate in the *Lα2* loop, as proven by a RSA below 5% for Q23 during the whole simulation, very close to what obtained in the case of MNEI-GLH. A similar situation is also observed in the simulations at 473 K. Although MNEI-E23Q would not be affected by water penetration in loop *Lα2*, these data suggest that, despite increased molecular motions, the structure remains steadily in place, retarding unfolding. This situation is described in Fig 7, in which is reported the distribution of the RSA for residue 23 over the three simulations in the mutant and the parent protein in both protonation states, providing an indication of water penetration and flexibility of the hydrophobic pocket.

**Table 2:** Hydrogen bonds from MNEI-E23Q simulations.

	E23Q1	E23Q2	E23Q3
**GLN23(H**_**ε2**_**)—GLY30(O)**	*92*.*5*	*95*.*0*	*92*.*3*
**TYR29(H)—GLN23(O**_**ε1**_**)**	*80*.*9*	*92*.*2*	*83*.*3*
**GLN28(H**_**ε2**_**)—GLN23(O**_**ε1**_**)**	*36*.*7*	*29*.*3*	*28*.*3*
**LEU32(H)—ASN24(O**_**δ1**_**)**	*82*.*9*	*87*.*0*	*41*.*9*
**ASN24(H**_**δ**_**)—LEU32(O)**	*83*.*7*		*49*.*2*
**ARG31(H**_**η1**_**)—ASN24(O)**			*20*.*2*
**THR12(H**_**γ1**_**)—ASP7(O)**	*13*.*2*	*78*.*6*	*94*.*5*
**GLY9(H)—THR12(O**_**γ1**_**)**	*83*.*0*	*11*.*9*	
**ARG84(H**_**η**_**)—ASN14(O**_**δ1**_**)**	*18*.*2*	*26*.*4*	*28*.*9*
**GLN13(H**_**ε**_**)—ILE8(O)**		*69*.*9*	*74*.*3*

Non-secondary structure H-bonds in the *Lα1* loop and percent occupancy from the three MD simulations at 300 K on MNEI-E23Q

### Thermal stability of MNEI-E23Q

**Fig 8:**
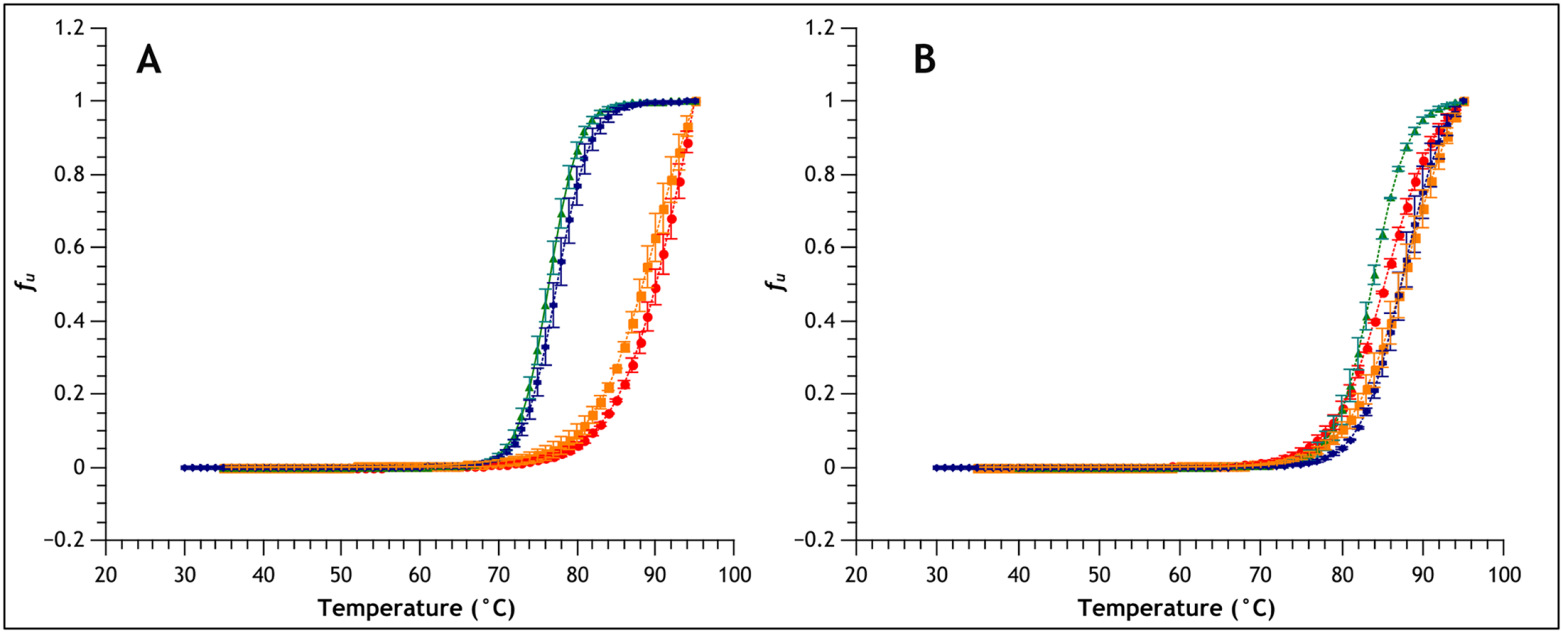
Thermal stability of MNEI and MNEI-E23Q. Comparison of CD unfolding curves for MNEI (A) and MNEI-E23Q (B) at pH 3.5 (red), 5.1 (orange), 6.8 (green) and 8.0 (blue). The image shows that melting temperature of MNEI drops about 15°C at neutral to alkaline pHs. On the contrary, MNEI-E23Q exhibits comparable stability to MNEI at acidic pH, and preserves this quality at neutral and alkaline pH.

In order to experimentally validate these theoretical results, we expressed and purified MNEI and MNEI-E23Q. The mutant shared the fold of the parent protein, as confirmed by Circular Dichroism spectroscopy (S6 Fig). CD spectroscopy was also used to monitor thermal denaturation and compare the proteins stability. Buffers at four pH values were used, namely 3.5, 5.1, 6.8 and 8.0, and unfolding was monitored through the signal at 215 nm. Fig 8 shows the fraction of unfolded protein vs temperature for the parent protein (Fig 8A) and the mutant (Fig 8B) at the different pHs. Despite being very resistant to thermal denaturation in acidic conditions, at neutral to alkaline pH MNEI melting temperature decreases about 15°C compared to the maximum of 90° at pH 3.5. On the contrary, MNEI-E23Q, which exhibits comparable stability at acidic pH, preserves this characteristic also at pH 6 and above. Preliminary sensory evaluation showed that the the new construct has comparable sweetness with MNEI, indicating that the biological activity is not affected by the point mutation. A thorough evaluation of the taste profile of the new protein will be the object of future studies. The melting temperatures corresponding to the curves in Fig 8 are reported in Table 3.

**Table 3:** Thermal stability of MNEI and MNEI-E23Q at various pHs.

	MNEI	MNEI-E23Q
**pH 3.5**	*91*.*0*	*86*.*7*
**pH 5.1**	*90*.*2*	*88*.*2*
**pH 6.8**	*76*.*3*	*84*.*3*
**pH 8.0**	*77*.*0*	*88*.*0*

Experimental melting temperatures (°C) for MNEI and MNEI-E23Q from CD denaturation curves. All measurements are accurate ± 0.1°C.

## Discussion

The existence of a correlation between pH and stability is a common feature of proteins presenting charged residues in hydrophobic regions [37–40] In many cases, these residues are essential for the biological function of the protein in exam, nonetheless they introduce a structural fragility, which becomes even more crucial if said proteins have industrial potential. This is the case of monellin and its single chain derivatives: these proteins, that could ideally be employed as sugar replacers in low-calorie food and beverages, undergo denaturation, aggregation and consequent loss of activity in some of the conditions that could occur in industrial processes. In the case of MNEI, pH-related instability has been attributed to E23, a glutamic residue buried in a hydrophobic pocket at the C-terminal of the α-helix. The abrupt jump in pK_a_ (and protonation state) deriving from exposure to water of E23 side chain is responsible for protein destabilization at neutral to alkaline pHs [24,44]. We have tried to understand at the atomic level the consequences of the change in the protonation state of E23 by running MD simulations at 300 and 473 K. The simulations show that deprotonation of E23 causes an increase of the flexibility of loop *Lα2* and exposure of the side chain to the bulk of the solvent, attracting water molecules to stabilize the negative charge. Nonetheless, penetration of water does not immediately affect the overall protein fold, and the protein is stable at room temperature even when E23 side chain is ionized, thanks to water mediated interactions. Conventional MD simulations cannot capture changes in protonation states, therefore this constitutes a simplified description of a system that will likely switch from one state to the other depending on the external environment. Nonetheless, these two situations are indirectly confirmed also by the available structural data: NMR studies probing MNEI surface at pH 2.9 had in fact pointed out a compact structure, with no internal water, whereas diffraction data on crystals obtained at pH 5.6 indicate that a water molecule is stably buried in the *Lα2* loop [47,63]. Molecular dynamic simulations suggest that the flexibility of such loop is reduced when E23 is protonated, making it less likely for water molecules to penetrate in the hydrophobic cavity, and retaining a strong hydrogen bond zipper that confers MNEI its well known thermostability at low pH. When water penetrates the loop at neutral to alkaline pH, such hydrogen bond network is perturbed, and MNEI stability is reduced. This situation can be reversed by introducing a mutation that allows to recreate the same arrangement of stabilizing interactions in a pH independent fashion. Mutant MNEI-E23Q preserves indeed, at alkaline pH, the stability typically observed for MNEI in acidic conditions. Sweet proteins have been receiving increasing attention as the search for new sweeteners has become a trending topic for food and beverage industries. Compared to other synthetic sweeteners, in fact, they offer many advantages: their degradation follows the natural path of dietary proteins and does not lead to the accumulation of toxic by-products, hinting to safety for human use; their non-carbohydrate nature makes them suitable for use by people suffering from diabetes and other metabolic dysfunctions; their enormous sweetening power allows for the use of minimal quantities and they can be obtained in large quantities with recombinant technologies [1,83]. Moreover, comparative taste evaluations of MNEI and other common sweeteners have proven that MNEI has the taste profile that more closely resembles that of sucrose, being more palatable than sweeteners with a bitter after-taste [84]. Therefore, the structural advantage introduced by the mutation E23Q translates in an increase of the application potential of MNEI, as the mutant becomes more resistant to physical stressors that could be encountered in industrial processes.

## Supporting Information

S1 Fig RMSD plots from MD runs. Simulations were run at 300 K (A) and 473 K (B). Black, MNEI-GLU_1; red, MNEI-GLU_2; green, MNEI-GLU_3; blue, MNEI-GLH_1; cyan, MNEI-GLH_2; magenta, MNEI-GLH_3.
(TIF)

S2 Fig Secondary structure (DSSP) plots of MD simulations at 300 K. (TIF)

S3 Fig Secondary structure (DSSP) plots of MD simulations at 473 K. (TIF)

S4 Fig Plot of the relative surface accessibility over time for MNEI. RSA was calculated from trajectories at 300 K (A) and 473 K (B). Black, MNEI-GLU_1; red, MNEI-GLU_2; green, MNEI-GLU_3; blue, MNEI-GLH_1; cyan, MNEI-GLH_2; magenta, MNEI-GLH_3.
(TIF)

S5 Fig Plot of the relative surface accessibility over time for MNEI-E23Q. RSA was calculated from trajectories at 300 K (A) and 473 K (B). Pink, MNEI-E23Q_1; dark green, MNEI-E23Q_2; orange, MNEI-E23Q_3.
(TIF)

S6 Fig Comparison of the CD spectra of the two proteins. Spectra were acquired on MNEI (red) and MNEI-E23Q (black) at pH 6.8.
(TIFF)
