# Supplementary figures and images for: Molecular Dynamics Driven Design of pH-Stabilized Mutants of MNEI, a Sweet Protein
Source: PLoS One. 2016 Jun 24;11(6):e0158372. doi: 10.1371/journal.pone.0158372 (PMC4920389; doi:10.1371/journal.pone.0158372)

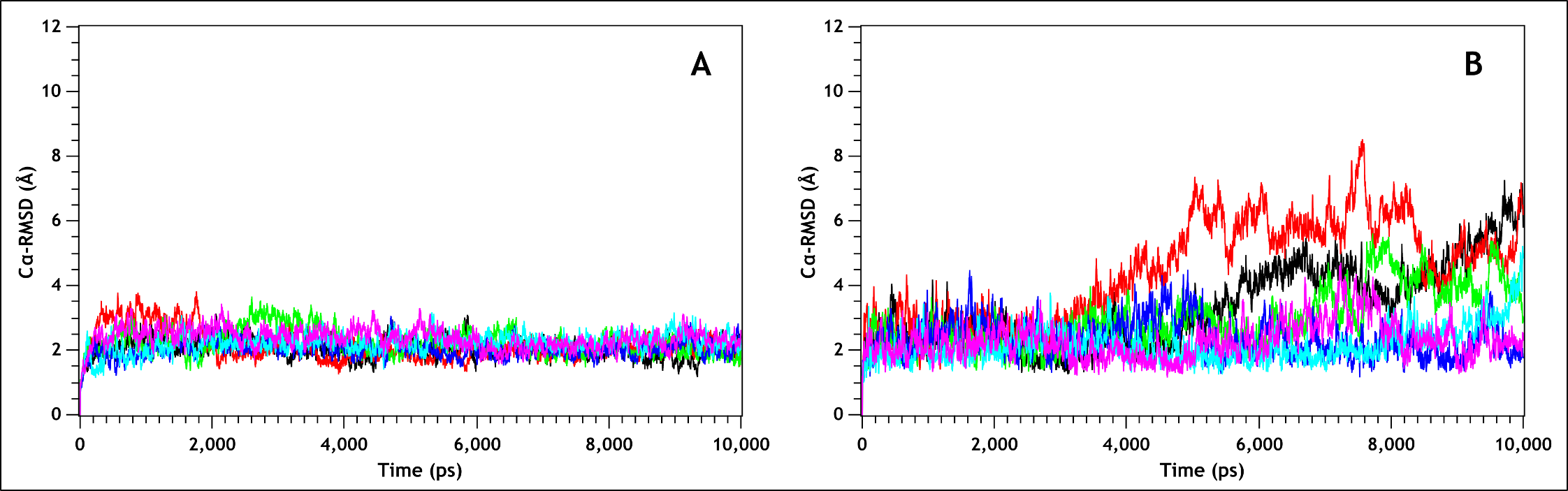

Supplement: S1 Fig — Simulations were run at 300 K (A) and 473 K (B). Black, MNEI-GLU_1; red, MNEI-GLU_2; green, MNEI-GLU_3; blue, MNEI-GLH_1; cyan, MNEI-GLH_2; magenta, MNEI-GLH_3. (TIF) [file pone.0158372.s001.tif]

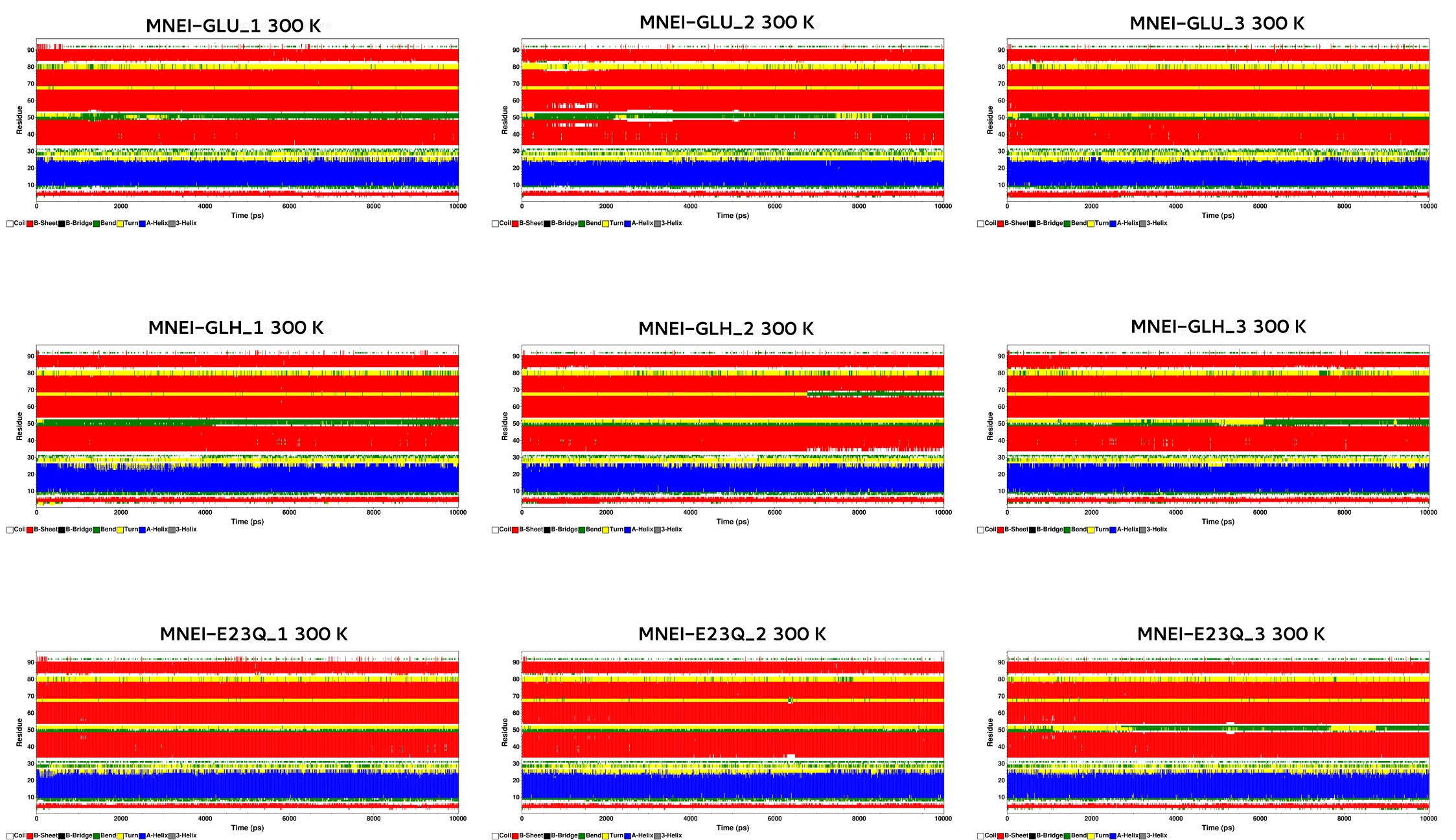

Supplement: S2 Fig — (TIF) [file pone.0158372.s002.tif]

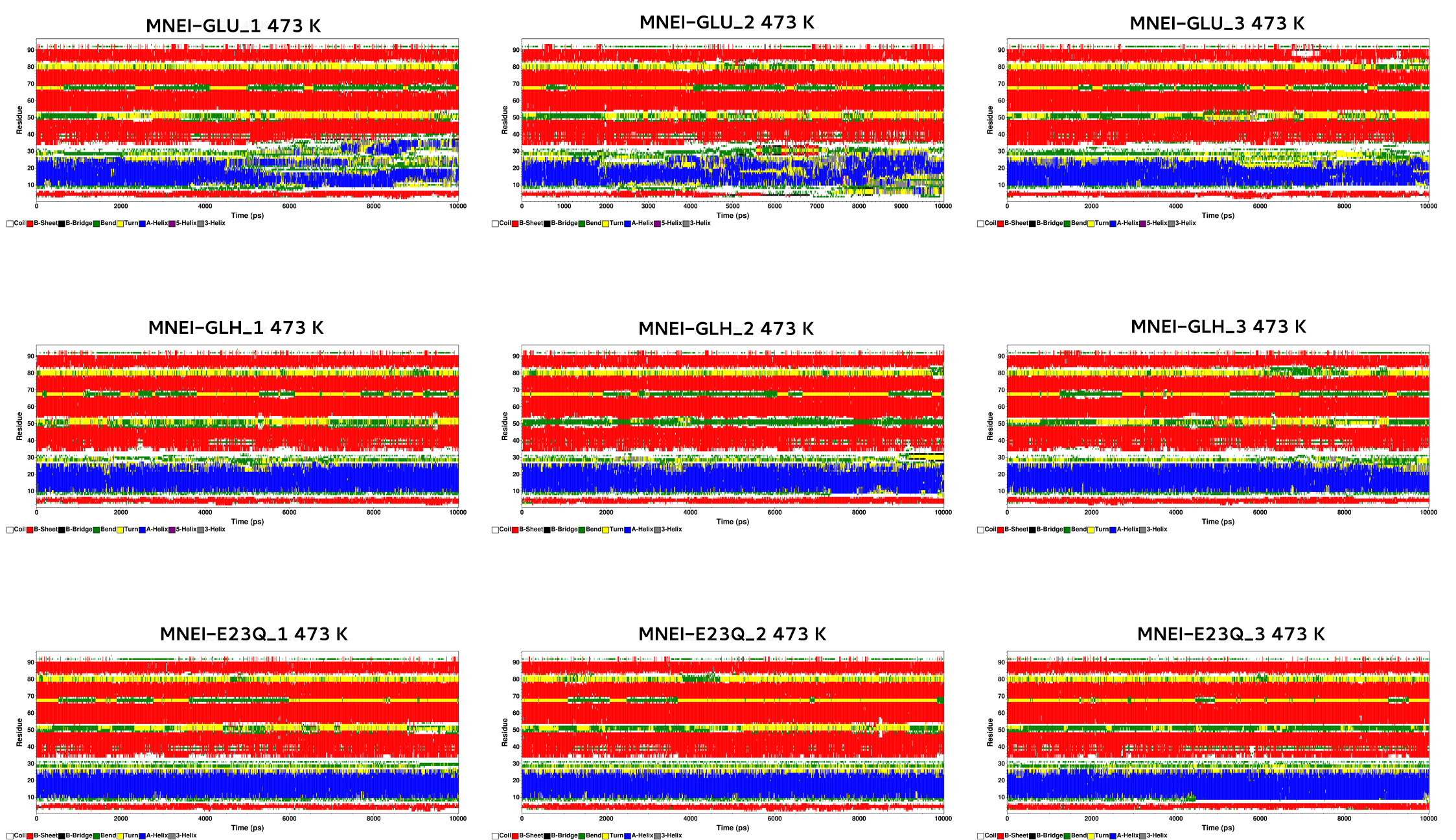

Supplement: S3 Fig — (TIF) [file pone.0158372.s003.tif]

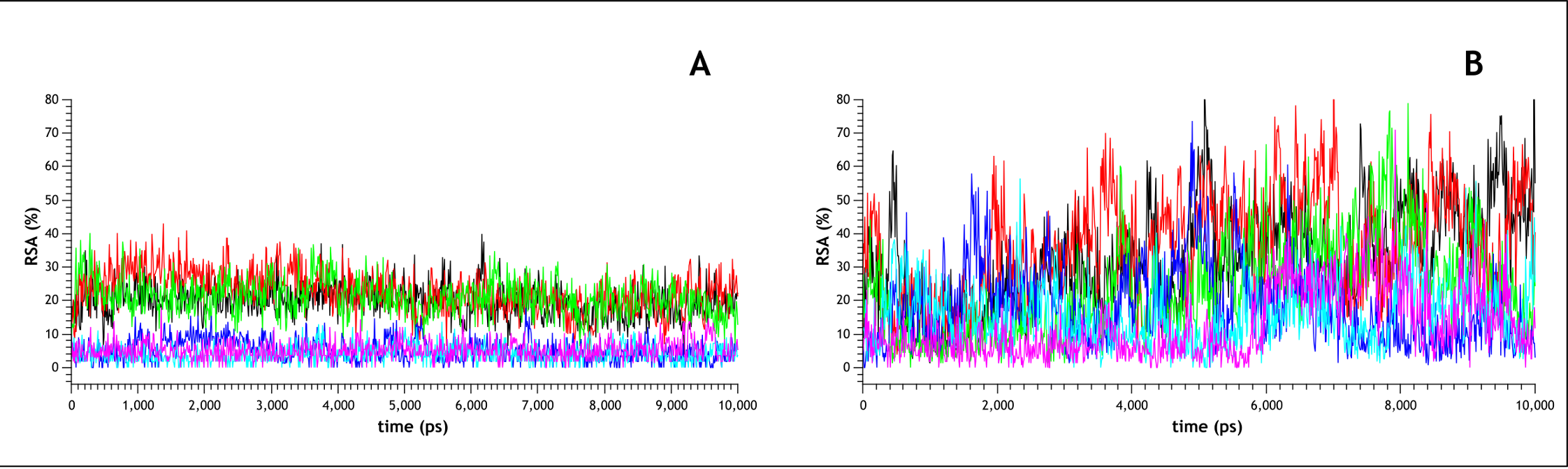

Supplement: S4 Fig — RSA was calculated from trajectories at 300 K (A) and 473 K (B). Black, MNEI-GLU_1; red, MNEI-GLU_2; green, MNEI-GLU_3; blue, MNEI-GLH_1; cyan, MNEI-GLH_2; magenta, MNEI-GLH_3. (TIF) [file pone.0158372.s004.tif]

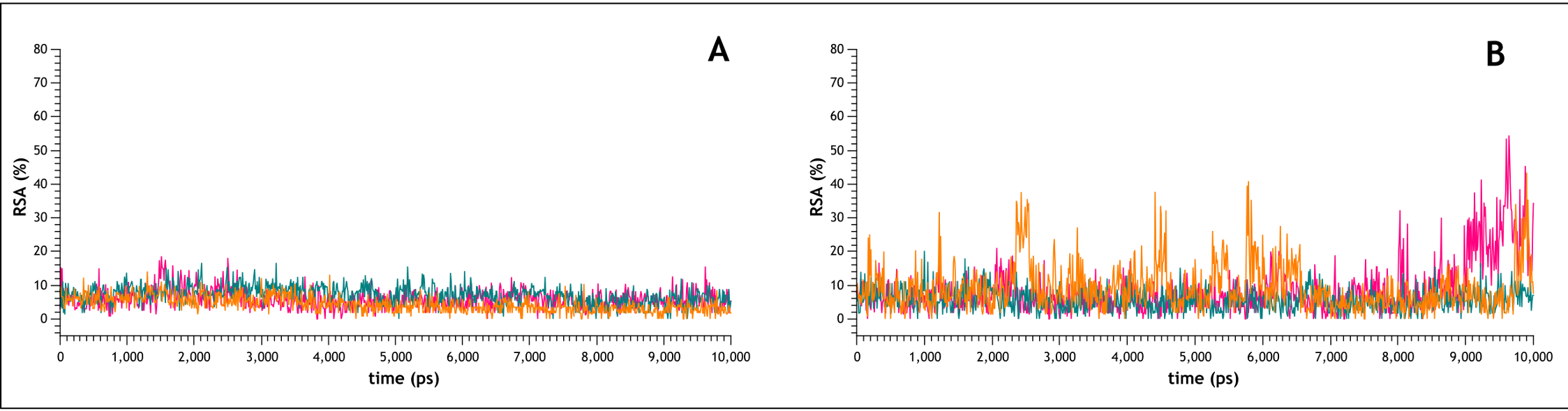

Supplement: S5 Fig — RSA was calculated from trajectories at 300 K (A) and 473 K (B). Pink, MNEI-E23Q_1; dark green, MNEI-E23Q_2; orange, MNEI-E23Q_3. (TIF) [file pone.0158372.s005.tif]

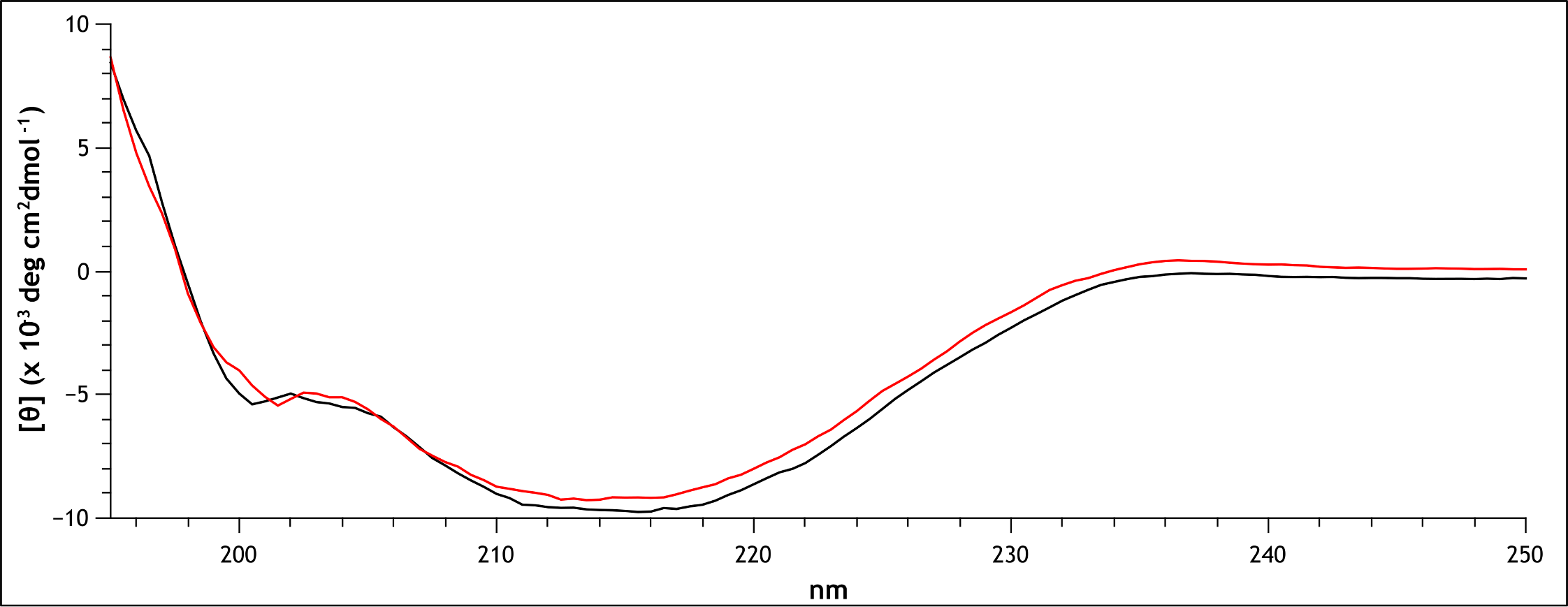

Supplement: S6 Fig — Spectra were acquired on MNEI (red) and MNEI-E23Q (black) at pH 6.8. (TIFF) [file pone.0158372.s006.tiff]
